# (1S*,4aR*,5S*,6S*,8aR*)-3-Benzyl-1-methyl-5,6-diphenyl-3,4,4a,5,6,8a-hexa­hydro-1*H*-2,3-benzoxazin-4-one

**DOI:** 10.1107/S1600536809044195

**Published:** 2009-10-31

**Authors:** Yan Wang, Jin-Long Wu

**Affiliations:** a Laboratory of Asymmetric Catalysis and Synthesis, Department of Chemistry, Zhejiang University, Hangzhou, Zhejiang 310027, People’s Republic of China

## Abstract

In the title compound, C_28_H_27_NO_2_, the oxazinone ring adopts a twist-boat conformation and the cyclo­hexene ring has a twisted envelope conformation. The crystal structure is stabilized by weak non-classical inter­molecular C—H⋯O hydrogen bonds.

## Related literature

For the synthesis of 1*H*-benzo[*d*][1,2]oxazin-4-ones by intra­molecular Diels–Alder (IMDA) cyclo­addition, see: Ishikawa *et al.* (2001 ▶). For microwave-assisted IMDA cyclo­addition, see: Dai & Shi (2007 ▶). For cyclo­addition of ester-tethered 1,3,8-nona­trienes, see: Wu *et al.* (2006 ▶), of sorbate-related 1,3,8-nona­trienes, see: Wu *et al.* (2007 ▶) and of hydroxamate-tethered 1,3,9-deca­trienes, see: Wang *et al.* (2009 ▶).
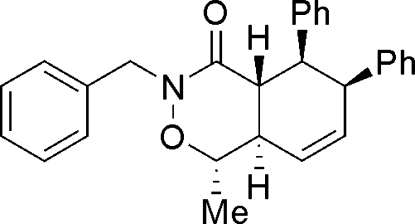


## Experimental

### 

#### Crystal data

C_28_H_27_NO_2_
*M* *_r_* = 409.51
Triclinic, 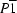

*a* = 7.9721 (5) Å
*b* = 11.0649 (7) Å
*c* = 13.578 (1) Å
α = 78.168 (2)°
β = 73.178 (2)°
γ = 82.819 (1)°
*V* = 1119.35 (13) Å^3^
*Z* = 2
Mo *K*α radiation
μ = 0.08 mm^−1^
*T* = 296 K
0.41 × 0.22 × 0.20 mm

#### Data collection

Rigaku R-AXIS RAPID diffractometer
Absorption correction: multi-scan (*ABSCOR*; Higashi, 1995 ▶) *T*_min_ = 0.970, *T*_max_ = 0.985
8442 measured reflections
3780 independent reflections
2761 reflections with *I* > 2σ(*I*)
*R*_int_ = 0.021

#### Refinement

*R*[*F* ^2^ > 2σ(*F* ^2^)] = 0.041
*wR*(*F* ^2^) = 0.113
*S* = 1.00
3780 reflections
282 parameters
H-atom parameters constrained
Δρ_max_ = 0.14 e Å^−3^
Δρ_min_ = −0.16 e Å^−3^

### 

Data collection: *PROCESS-AUTO* (Rigaku, 2006 ▶); cell refinement: *PROCESS-AUTO*; data reduction: *CrystalStructure* (Rigaku/MSC, 2007 ▶); program(s) used to solve structure: *SHELXS97* (Sheldrick, 2008 ▶); program(s) used to refine structure: *SHELXL97* (Sheldrick, 2008 ▶); molecular graphics: *ORTEP-3 for Windows* (Farrugia, 1997 ▶); software used to prepare material for publication: *WinGX* (Farrugia, 1999 ▶).

## Supplementary Material

Crystal structure: contains datablocks global, I. DOI: 10.1107/S1600536809044195/lx2119sup1.cif

Structure factors: contains datablocks I. DOI: 10.1107/S1600536809044195/lx2119Isup2.hkl

Additional supplementary materials:  crystallographic information; 3D view; checkCIF report

## Figures and Tables

**Table 1:** Hydrogen-bond geometry (Å, °)

*D*—H⋯*A*	*D*—H	H⋯*A*	*D*⋯*A*	*D*—H⋯*A*
C6—H6⋯O1^i^	0.93	2.58	3.311 (2)	135
C7—H7⋯O1^ii^	0.98	2.47	3.378 (2)	154

Symmetry codes: (i) 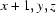; (ii) 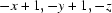.

**Fig. 1.**
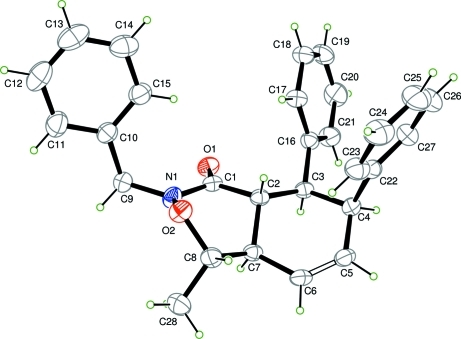
The molecular structure of the title compound with the atom numbering scheme. Displacement ellipsoids are drawn at 40% probability level. H atoms are presented as a small spheres of arbitrary radius.

**Fig. 2.**
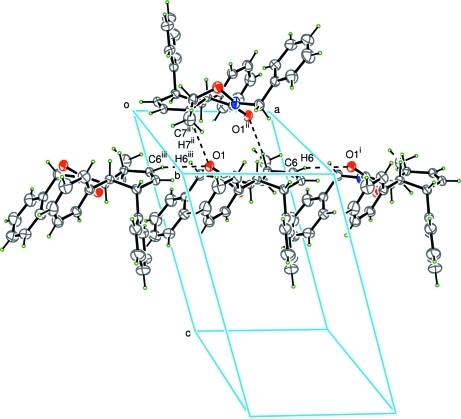
C—H···O interactions (dotted lines) in the crystal structure of the title compound. [Symmetry codes: (i) x + 1, y, z; (ii) -x + 1, - y + 1, - z; (iii) x - 1, y, z].

## supplementary crystallographic information

### Comment

The title compound is a derivative of 1*H*-benzo[*d*][1,2]oxazin-4-ones which have been prepared by intramolecular Diels-Alder (IMDA) cycloaddition of the hydroxamate-tethered 1,3,9-decatrienes (Ishikawa *et al.*, 2001). In our previous work on microwave-assisted IMDA cycloadditions (Dai *et al.*, 2007), we have investigated the ester-tethered 1,3,8-nonatrienes (Wu *et al.*, 2006), the sorbate-related 1,3,8-nonatrienes (Wu *et al.*, 2007), and the hydroxamate-tethered 1,3,9-decatrienes (Wang *et al.*, 2009). When racemic (3*E*,5*E*)-6-phenylhexa-3,5-dien-2-yl *N*-benzyl-cinnamoylhydroxamate was heated under microwave irradiation the title compound, together with another two major stereomers, was formed. Here we report the crystal structure of title compound (Fig. 1).

In the crystal structure of the title compound, there are one oxazinone ring and one cyclohexene ring. The oxazinone ring C1—C2/C7—C8/O2—N1 adopts a twist-boat conformation, whereas the cyclohexene ring C2—C7 has a twisted envelope conformation. Bond length of C3—C4 is larger than normal C—C single bond because of the hindrance between two phenyl rings at C3 and C4. The crystal packing (Fig. 2) is stabilized by weak non-classical intermolecular C—H···O hydrogen bonds; the first between an H atom of the cyclohexene ring and the oxygen of the CO unit, with a C6—H6···O1^i^, the second between an H atom of the ringjunction carbon and the oxygen of the CO unit, with a C7—H7···O1^ii^, respectively (Table 1).

### Experimental

To a 10 ml pressurized process vial was added racemic (3*E*,5*E*)-6-phenylhexa-3,5-dien-2-yl *N*-benzyl-cinnamoylhydroxamate (93.0 mg, 0.23 mmol) and MeCN (5 ml). The loaded vial was then sealed with a cap containing a silicon septum, and put into the microwave cavity and heated at 453 K for 30 min (the holding time) with the temperature measured by an IR sensor. After cooling to room temperature, the reaction mixture was concentrated under reduced pressure and the residue was then purified by colum chromatography (silica gel, 5% EtOAc in petroleum ether) to give the title compound in 10% yield (9.0 mg) as a white solid, and another two stereomers (in 29% and 49% yield, respectively). For the title compound, m.p. 464–466 K (EtOAc-hexane). Single crystals suitable for X-ray diffraction of the title compound were grown in the mixed solvent of ethyl acetate and hexane.

### Refinement

The H atoms were placed in calculated positions with C—H = 0.93–0.98 Å, and included in the refinement in riding model, with *U*_iso_(H) = 1.2*U*_eq_ (carrier atom).

### Crystal data

**Table:**

C_28_H_27_NO_2_	*Z* = 2
*M**_r_* = 409.51	*F*(000) = 436
Triclinic, *P*1	*D*_x_ = 1.215 Mg m^−^^3^
Hall symbol: -P 1	Mo *K*α radiation, λ = 0.71073 Å
*a* = 7.9721 (5) Å	Cell parameters from 6812 reflections
*b* = 11.0649 (7) Å	θ = 3.0–27.4°
*c* = 13.578 (1) Å	µ = 0.08 mm^−^^1^
α = 78.168 (2)°	*T* = 296 K
β = 73.178 (2)°	Block, colorless
γ = 82.819 (1)°	0.41 × 0.22 × 0.20 mm
*V* = 1119.35 (13) Å^3^	

### Data collection

**Table:**

Rigaku R-AXIS RAPID diffractometer	3780 independent reflections
Radiation source: rolling anode	2761 reflections with *I* > 2σ(*I*)
graphite	*R*_int_ = 0.021
Detector resolution: 10.00 pixels mm^-1^	θ_max_ = 25.0°, θ_min_ = 3.0°
ω scans	*h* = −8→9
Absorption correction: multi-scan (*ABSCOR*; Higashi, 1995)	*k* = −13→13
*T*_min_ = 0.970, *T*_max_ = 0.985	*l* = −16→16
8442 measured reflections	

### Refinement

**Table:**

Refinement on *F*^2^	Secondary atom site location: difference Fourier map
Least-squares matrix: full	Hydrogen site location: inferred from neighbouring sites
*R*[*F*^2^ > 2σ(*F*^2^)] = 0.041	H-atom parameters constrained
*wR*(*F*^2^) = 0.113	*w* = 1/[σ^2^(*F*_o_^2^) + (0.035*P*)^2^ + 0.520*P*] where *P* = (*F*_o_^2^ + 2*F*_c_^2^)/3
*S* = 1.00	(Δ/σ)_max_ < 0.001
3780 reflections	Δρ_max_ = 0.14 e Å^−^^3^
282 parameters	Δρ_min_ = −0.16 e Å^−^^3^
0 restraints	Extinction correction: *SHELXL97* (Sheldrick, 2008), Fc*=kFc[1+0.001xFc^2^λ^3^/sin(2θ)]^-1/4^
Primary atom site location: structure-invariant direct methods	Extinction coefficient: 0.029 (2)

### Special details

**Table:**

Geometry. All e.s.d.'s (except the e.s.d. in the dihedral angle between two l.s. planes) are estimated using the full covariance matrix. The cell e.s.d.'s are taken into account individually in the estimation of e.s.d.'s in distances, angles and torsion angles; correlations between e.s.d.'s in cell parameters are only used when they are defined by crystal symmetry. An approximate (isotropic) treatment of cell e.s.d.'s is used for estimating e.s.d.'s involving l.s. planes.
Refinement. Refinement of *F*^2^ against ALL reflections. The weighted *R*-factor *wR* and goodness of fit *S* are based on *F*^2^, conventional *R*-factors *R* are based on *F*, with *F* set to zero for negative *F*^2^. The threshold expression of *F*^2^ > σ(*F*^2^) is used only for calculating *R*-factors(gt) *etc*. and is not relevant to the choice of reflections for refinement. *R*-factors based on *F*^2^ are statistically about twice as large as those based on *F*, and *R*- factors based on ALL data will be even larger.

### Fractional atomic coordinates and isotropic or equivalent isotropic displacement parameters (Å^2^)

**Table:**

	*x*	*y*	*z*	*U*_iso_*/*U*_eq_	
O1	0.31521 (17)	0.51064 (13)	0.10452 (11)	0.0527 (4)	
O2	0.52969 (18)	0.30895 (13)	0.27091 (11)	0.0566 (4)	
N1	0.4262 (2)	0.34429 (15)	0.19925 (14)	0.0521 (4)	
C1	0.4170 (2)	0.46717 (18)	0.15722 (14)	0.0430 (5)	
C2	0.5572 (2)	0.53344 (17)	0.17483 (14)	0.0398 (4)	
H2	0.5323	0.5327	0.2500	0.048*	
C3	0.5745 (2)	0.66685 (17)	0.11789 (14)	0.0423 (4)	
H3	0.6124	0.6640	0.0429	0.051*	
C4	0.7261 (2)	0.72170 (18)	0.14317 (15)	0.0477 (5)	
H4	0.7563	0.7967	0.0909	0.057*	
C5	0.8884 (2)	0.6345 (2)	0.12960 (15)	0.0516 (5)	
H5	0.9952	0.6667	0.1207	0.062*	
C6	0.8889 (2)	0.5157 (2)	0.12950 (15)	0.0487 (5)	
H6	0.9953	0.4685	0.1222	0.058*	
C7	0.7277 (2)	0.45197 (17)	0.14044 (15)	0.0425 (4)	
H7	0.7338	0.4351	0.0714	0.051*	
C8	0.7139 (3)	0.32775 (19)	0.21604 (17)	0.0522 (5)	
H8	0.7741	0.3327	0.2685	0.063*	
C9	0.2941 (3)	0.2601 (2)	0.21231 (18)	0.0568 (6)	
H9A	0.3504	0.1772	0.2120	0.068*	
H9B	0.2461	0.2817	0.1525	0.068*	
C10	0.1442 (3)	0.2581 (2)	0.31043 (16)	0.0527 (5)	
C11	0.0603 (3)	0.1503 (2)	0.3554 (2)	0.0759 (7)	
H11	0.1029	0.0782	0.3286	0.091*	
C12	−0.0877 (4)	0.1488 (3)	0.4408 (2)	0.0945 (9)	
H12	−0.1452	0.0763	0.4694	0.113*	
C13	−0.1489 (4)	0.2533 (4)	0.4827 (2)	0.0920 (9)	
H13	−0.2469	0.2519	0.5402	0.110*	
C14	−0.0652 (3)	0.3601 (3)	0.4396 (2)	0.0801 (8)	
H14	−0.1060	0.4312	0.4683	0.096*	
C15	0.0799 (3)	0.3628 (2)	0.35354 (18)	0.0649 (6)	
H15	0.1347	0.4362	0.3243	0.078*	
C16	0.4076 (2)	0.75084 (17)	0.13603 (15)	0.0453 (5)	
C17	0.2782 (3)	0.7394 (2)	0.23037 (17)	0.0536 (5)	
H17	0.2919	0.6763	0.2850	0.064*	
C18	0.1290 (3)	0.8199 (2)	0.2451 (2)	0.0653 (6)	
H18	0.0439	0.8107	0.3091	0.078*	
C19	0.1071 (3)	0.9132 (2)	0.1653 (2)	0.0748 (7)	
H19	0.0068	0.9672	0.1748	0.090*	
C20	0.2332 (4)	0.9266 (2)	0.0717 (2)	0.0797 (8)	
H20	0.2187	0.9901	0.0175	0.096*	
C21	0.3824 (3)	0.8461 (2)	0.05714 (19)	0.0632 (6)	
H21	0.4672	0.8564	−0.0069	0.076*	
C22	0.6757 (3)	0.7609 (2)	0.24998 (17)	0.0519 (5)	
C23	0.6937 (3)	0.6795 (2)	0.33864 (18)	0.0639 (6)	
H23	0.7340	0.5977	0.3341	0.077*	
C24	0.6525 (4)	0.7179 (3)	0.4344 (2)	0.0852 (8)	
H24	0.6639	0.6617	0.4936	0.102*	
C25	0.5950 (4)	0.8386 (4)	0.4419 (3)	0.1007 (11)	
H25	0.5697	0.8649	0.5058	0.121*	
C26	0.5751 (4)	0.9202 (3)	0.3550 (3)	0.0956 (10)	
H26	0.5346	1.0019	0.3600	0.115*	
C27	0.6148 (3)	0.8819 (2)	0.2602 (2)	0.0708 (7)	
H27	0.6006	0.9383	0.2017	0.085*	
C28	0.8005 (3)	0.2201 (2)	0.1641 (2)	0.0756 (7)	
H28A	0.7915	0.1457	0.2156	0.091*	
H28B	0.9221	0.2336	0.1308	0.091*	
H28C	0.7434	0.2121	0.1127	0.091*	

### Atomic displacement parameters (Å^2^)

**Table:**

	*U*^11^	*U*^22^	*U*^33^	*U*^12^	*U*^13^	*U*^23^
O1	0.0413 (7)	0.0654 (9)	0.0550 (9)	−0.0001 (7)	−0.0218 (7)	−0.0085 (7)
O2	0.0507 (8)	0.0596 (9)	0.0558 (9)	−0.0015 (7)	−0.0195 (7)	0.0036 (7)
N1	0.0412 (9)	0.0519 (10)	0.0642 (11)	−0.0073 (8)	−0.0211 (8)	−0.0006 (8)
C1	0.0341 (9)	0.0524 (12)	0.0409 (11)	0.0023 (9)	−0.0082 (8)	−0.0106 (9)
C2	0.0350 (9)	0.0482 (11)	0.0372 (10)	0.0006 (8)	−0.0114 (8)	−0.0099 (8)
C3	0.0393 (10)	0.0500 (11)	0.0366 (10)	−0.0024 (9)	−0.0083 (8)	−0.0083 (8)
C4	0.0430 (11)	0.0506 (11)	0.0469 (12)	−0.0077 (9)	−0.0058 (9)	−0.0096 (9)
C5	0.0352 (10)	0.0675 (14)	0.0532 (13)	−0.0053 (10)	−0.0067 (9)	−0.0196 (10)
C6	0.0332 (10)	0.0633 (13)	0.0501 (12)	0.0055 (9)	−0.0101 (8)	−0.0184 (10)
C7	0.0360 (10)	0.0511 (11)	0.0441 (11)	0.0010 (9)	−0.0137 (8)	−0.0156 (9)
C8	0.0436 (11)	0.0542 (12)	0.0620 (13)	0.0027 (10)	−0.0221 (10)	−0.0100 (10)
C9	0.0505 (12)	0.0551 (13)	0.0665 (14)	−0.0087 (10)	−0.0123 (10)	−0.0165 (11)
C10	0.0487 (12)	0.0586 (13)	0.0517 (13)	−0.0069 (10)	−0.0165 (10)	−0.0055 (10)
C11	0.0760 (17)	0.0662 (16)	0.0793 (18)	−0.0168 (13)	−0.0170 (14)	0.0020 (13)
C12	0.087 (2)	0.101 (2)	0.079 (2)	−0.0365 (18)	−0.0093 (17)	0.0198 (17)
C13	0.0741 (18)	0.139 (3)	0.0548 (17)	−0.019 (2)	−0.0074 (13)	−0.0057 (18)
C14	0.0695 (16)	0.110 (2)	0.0634 (16)	−0.0016 (16)	−0.0143 (13)	−0.0298 (15)
C15	0.0595 (14)	0.0739 (16)	0.0606 (15)	−0.0108 (12)	−0.0099 (11)	−0.0159 (12)
C16	0.0440 (11)	0.0445 (11)	0.0491 (12)	−0.0005 (9)	−0.0153 (9)	−0.0098 (9)
C17	0.0474 (11)	0.0563 (12)	0.0544 (13)	0.0043 (10)	−0.0104 (10)	−0.0135 (10)
C18	0.0496 (13)	0.0676 (15)	0.0789 (17)	0.0035 (12)	−0.0091 (11)	−0.0298 (13)
C19	0.0594 (15)	0.0570 (15)	0.113 (2)	0.0169 (12)	−0.0304 (15)	−0.0284 (15)
C20	0.0811 (18)	0.0561 (15)	0.096 (2)	0.0101 (14)	−0.0330 (16)	0.0051 (13)
C21	0.0598 (14)	0.0567 (13)	0.0653 (15)	0.0028 (12)	−0.0150 (11)	−0.0002 (11)
C22	0.0401 (10)	0.0617 (13)	0.0571 (13)	−0.0076 (10)	−0.0083 (9)	−0.0228 (10)
C23	0.0598 (14)	0.0803 (16)	0.0576 (14)	−0.0005 (12)	−0.0188 (11)	−0.0240 (12)
C24	0.0789 (18)	0.124 (2)	0.0601 (16)	−0.0048 (17)	−0.0195 (13)	−0.0333 (16)
C25	0.093 (2)	0.139 (3)	0.084 (2)	−0.013 (2)	−0.0077 (18)	−0.071 (2)
C26	0.097 (2)	0.090 (2)	0.104 (2)	−0.0098 (18)	−0.0022 (19)	−0.059 (2)
C27	0.0675 (15)	0.0639 (15)	0.0812 (18)	−0.0098 (12)	−0.0069 (13)	−0.0299 (13)
C28	0.0642 (15)	0.0576 (14)	0.107 (2)	0.0089 (12)	−0.0245 (14)	−0.0250 (14)

### Geometric parameters (Å, °)

**Table:**

O1—C1	1.224 (2)	C12—H12	0.9300
O2—N1	1.417 (2)	C13—C14	1.368 (4)
O2—C8	1.461 (2)	C13—H13	0.9300
N1—C1	1.364 (2)	C14—C15	1.385 (3)
N1—C9	1.441 (3)	C14—H14	0.9300
C1—C2	1.508 (3)	C15—H15	0.9300
C2—C3	1.522 (3)	C16—C21	1.381 (3)
C2—C7	1.542 (2)	C16—C17	1.386 (3)
C2—H2	0.9800	C17—C18	1.385 (3)
C3—C16	1.513 (3)	C17—H17	0.9300
C3—C4	1.567 (3)	C18—C19	1.370 (3)
C3—H3	0.9800	C18—H18	0.9300
C4—C5	1.504 (3)	C19—C20	1.367 (4)
C4—C22	1.526 (3)	C19—H19	0.9300
C4—H4	0.9800	C20—C21	1.385 (3)
C5—C6	1.315 (3)	C20—H20	0.9300
C5—H5	0.9300	C21—H21	0.9300
C6—C7	1.497 (3)	C22—C23	1.380 (3)
C6—H6	0.9300	C22—C27	1.386 (3)
C7—C8	1.533 (3)	C23—C24	1.387 (3)
C7—H7	0.9800	C23—H23	0.9300
C8—C28	1.500 (3)	C24—C25	1.371 (4)
C8—H8	0.9800	C24—H24	0.9300
C9—C10	1.509 (3)	C25—C26	1.367 (4)
C9—H9A	0.9700	C25—H25	0.9300
C9—H9B	0.9700	C26—C27	1.375 (4)
C10—C11	1.381 (3)	C26—H26	0.9300
C10—C15	1.380 (3)	C27—H27	0.9300
C11—C12	1.393 (4)	C28—H28A	0.9600
C11—H11	0.9300	C28—H28B	0.9600
C12—C13	1.367 (4)	C28—H28C	0.9600
			
N1—O2—C8	109.53 (14)	C13—C12—C11	120.3 (3)
C1—N1—O2	115.87 (15)	C13—C12—H12	119.9
C1—N1—C9	125.17 (16)	C11—C12—H12	119.9
O2—N1—C9	114.00 (16)	C12—C13—C14	119.7 (3)
O1—C1—N1	121.51 (18)	C12—C13—H13	120.2
O1—C1—C2	126.71 (18)	C14—C13—H13	120.2
N1—C1—C2	111.46 (15)	C13—C14—C15	120.3 (3)
C1—C2—C3	115.43 (15)	C13—C14—H14	119.9
C1—C2—C7	104.30 (14)	C15—C14—H14	119.9
C3—C2—C7	111.71 (15)	C10—C15—C14	121.0 (2)
C1—C2—H2	108.4	C10—C15—H15	119.5
C3—C2—H2	108.4	C14—C15—H15	119.5
C7—C2—H2	108.4	C21—C16—C17	117.39 (19)
C16—C3—C2	115.82 (15)	C21—C16—C3	119.58 (18)
C16—C3—C4	111.53 (15)	C17—C16—C3	123.01 (17)
C2—C3—C4	109.12 (15)	C18—C17—C16	121.5 (2)
C16—C3—H3	106.6	C18—C17—H17	119.2
C2—C3—H3	106.6	C16—C17—H17	119.2
C4—C3—H3	106.6	C19—C18—C17	119.9 (2)
C5—C4—C22	111.07 (17)	C19—C18—H18	120.1
C5—C4—C3	110.91 (16)	C17—C18—H18	120.1
C22—C4—C3	114.42 (15)	C20—C19—C18	119.7 (2)
C5—C4—H4	106.6	C20—C19—H19	120.1
C22—C4—H4	106.6	C18—C19—H19	120.1
C3—C4—H4	106.6	C19—C20—C21	120.3 (2)
C6—C5—C4	124.13 (18)	C19—C20—H20	119.8
C6—C5—H5	117.9	C21—C20—H20	119.8
C4—C5—H5	117.9	C16—C21—C20	121.2 (2)
C5—C6—C7	123.75 (18)	C16—C21—H21	119.4
C5—C6—H6	118.1	C20—C21—H21	119.4
C7—C6—H6	118.1	C23—C22—C27	117.6 (2)
C6—C7—C8	113.55 (15)	C23—C22—C4	121.82 (19)
C6—C7—C2	112.49 (16)	C27—C22—C4	120.5 (2)
C8—C7—C2	108.48 (15)	C22—C23—C24	121.0 (2)
C6—C7—H7	107.3	C22—C23—H23	119.5
C8—C7—H7	107.3	C24—C23—H23	119.5
C2—C7—H7	107.3	C25—C24—C23	120.1 (3)
O2—C8—C28	111.25 (18)	C25—C24—H24	120.0
O2—C8—C7	109.67 (15)	C23—C24—H24	120.0
C28—C8—C7	113.35 (19)	C26—C25—C24	119.7 (3)
O2—C8—H8	107.4	C26—C25—H25	120.2
C28—C8—H8	107.4	C24—C25—H25	120.2
C7—C8—H8	107.4	C25—C26—C27	120.2 (3)
N1—C9—C10	115.45 (18)	C25—C26—H26	119.9
N1—C9—H9A	108.4	C27—C26—H26	119.9
C10—C9—H9A	108.4	C26—C27—C22	121.4 (3)
N1—C9—H9B	108.4	C26—C27—H27	119.3
C10—C9—H9B	108.4	C22—C27—H27	119.3
H9A—C9—H9B	107.5	C8—C28—H28A	109.5
C11—C10—C15	118.3 (2)	C8—C28—H28B	109.5
C11—C10—C9	119.4 (2)	H28A—C28—H28B	109.5
C15—C10—C9	122.2 (2)	C8—C28—H28C	109.5
C10—C11—C12	120.6 (3)	H28A—C28—H28C	109.5
C10—C11—H11	119.7	H28B—C28—H28C	109.5
C12—C11—H11	119.7		
			
C8—O2—N1—C1	−66.3 (2)	N1—C9—C10—C11	−150.2 (2)
C8—O2—N1—C9	137.27 (17)	N1—C9—C10—C15	34.1 (3)
O2—N1—C1—O1	−171.05 (16)	C15—C10—C11—C12	1.3 (4)
C9—N1—C1—O1	−17.6 (3)	C9—C10—C11—C12	−174.5 (2)
O2—N1—C1—C2	15.0 (2)	C10—C11—C12—C13	−1.7 (4)
C9—N1—C1—C2	168.44 (18)	C11—C12—C13—C14	0.8 (5)
O1—C1—C2—C3	−1.8 (3)	C12—C13—C14—C15	0.5 (4)
N1—C1—C2—C3	171.77 (15)	C11—C10—C15—C14	−0.1 (3)
O1—C1—C2—C7	−124.77 (19)	C9—C10—C15—C14	175.7 (2)
N1—C1—C2—C7	48.8 (2)	C13—C14—C15—C10	−0.9 (4)
C1—C2—C3—C16	53.8 (2)	C2—C3—C16—C21	−148.07 (19)
C7—C2—C3—C16	172.73 (15)	C4—C3—C16—C21	86.3 (2)
C1—C2—C3—C4	−179.38 (15)	C2—C3—C16—C17	33.9 (3)
C7—C2—C3—C4	−60.46 (19)	C4—C3—C16—C17	−91.7 (2)
C16—C3—C4—C5	177.36 (16)	C21—C16—C17—C18	0.3 (3)
C2—C3—C4—C5	48.1 (2)	C3—C16—C17—C18	178.32 (19)
C16—C3—C4—C22	50.8 (2)	C16—C17—C18—C19	0.1 (3)
C2—C3—C4—C22	−78.5 (2)	C17—C18—C19—C20	−0.3 (4)
C22—C4—C5—C6	108.5 (2)	C18—C19—C20—C21	0.2 (4)
C3—C4—C5—C6	−19.9 (3)	C17—C16—C21—C20	−0.4 (3)
C4—C5—C6—C7	1.5 (3)	C3—C16—C21—C20	−178.5 (2)
C5—C6—C7—C8	−135.8 (2)	C19—C20—C21—C16	0.1 (4)
C5—C6—C7—C2	−12.1 (3)	C5—C4—C22—C23	−37.7 (3)
C1—C2—C7—C6	167.33 (15)	C3—C4—C22—C23	88.8 (2)
C3—C2—C7—C6	42.0 (2)	C5—C4—C22—C27	140.3 (2)
C1—C2—C7—C8	−66.20 (18)	C3—C4—C22—C27	−93.2 (2)
C3—C2—C7—C8	168.46 (15)	C27—C22—C23—C24	−0.1 (3)
N1—O2—C8—C28	−83.2 (2)	C4—C22—C23—C24	177.9 (2)
N1—O2—C8—C7	43.0 (2)	C22—C23—C24—C25	−0.8 (4)
C6—C7—C8—O2	145.60 (16)	C23—C24—C25—C26	1.3 (5)
C2—C7—C8—O2	19.7 (2)	C24—C25—C26—C27	−0.9 (5)
C6—C7—C8—C28	−89.4 (2)	C25—C26—C27—C22	−0.1 (4)
C2—C7—C8—C28	144.74 (17)	C23—C22—C27—C26	0.6 (4)
C1—N1—C9—C10	−84.5 (3)	C4—C22—C27—C26	−177.5 (2)
O2—N1—C9—C10	69.4 (2)		

### Hydrogen-bond geometry (Å, °)

**Table:**

*D*—H···*A*	*D*—H	H···*A*	*D*···*A*	*D*—H···*A*
C6—H6···O1^i^	0.93	2.58	3.311 (2)	135
C7—H7···O1^ii^	0.98	2.47	3.378 (2)	154

Symmetry codes: (i) *x*+1, *y*, *z*; (ii) −*x*+1, −*y*+1, −*z*.

## References

[bb1] Dai, W.-M. & Shi, J. (2007). *Comb. Chem. High Throughput Screening*, **10**, 837–856. 10.2174/138620707783220338 18288947

[bb2] Farrugia, L. J. (1997). *J. Appl. Cryst.* **30**, 565.

[bb3] Farrugia, L. J. (1999). *J. Appl. Cryst.* **32**, 837–838.

[bb4] Higashi, T. (1995). *ABSCOR* Rigaku Corporation, Tokyo, Japan.

[bb5] Ishikawa, T., Senzaki, M., Kadoya, R., Morimoto, T., Miyake, N., Izawa, M. & Saito, S. (2001). *J. Am. Chem. Soc.* **123**, 4607–4608. 10.1021/ja010083z 11457250

[bb6] Rigaku (2006). *PROCESS-AUTO* Rigaku Corporation, Tokyo, Japan.

[bb7] Rigaku/MSC (2007). *CrystalStructure* Rigaku/MSC, The Woodlands, Texas, USA.

[bb8] Sheldrick, G. M. (2008). *Acta Cryst.* A**64**, 112–122. 10.1107/S0108767307043930 18156677

[bb9] Wang, Y., Wu, J. & Dai, W.-M. (2009). *Synlett*, pp. 2862–2866.

[bb10] Wu, J., Sun, L. & Dai, W.-M. (2006). *Tetrahedron*, **62**, 8360–8372.

[bb11] Wu, J., Yu, H., Wang, Y., Xing, X. & Dai, W.-M. (2007). *Tetrahedron Lett. ***48**, 6543–6547.

